# Tissue Microstructure Is Linked to MRI Parameters and Metabolite Levels in Prostate Cancer

**DOI:** 10.3389/fonc.2016.00146

**Published:** 2016-06-14

**Authors:** Kirsten Margrete Selnæs, Riyas Vettukattil, Helena Bertilsson, Alan J. Wright, Arend Heerschap, Anders Angelsen, May-Britt Tessem, Tone Frost Bathen

**Affiliations:** ^1^Department of Circulation and Medical Imaging, Norwegian University of Science and Technology, Trondheim, Norway; ^2^St. Olavs Hospital, Trondheim, Norway; ^3^Department of Cancer Research and Molecular Medicine, Norwegian University of Science and Technology, Trondheim, Norway; ^4^Department of Urology, St. Olavs Hospital, Trondheim, Norway; ^5^Cancer Research UK Cambridge Institute, University of Cambridge, Cambridge, UK; ^6^Department of Radiology and Nuclear Medicine, Radboud University Medical Center, Nijmegen, Netherlands

**Keywords:** ADC, magnetic resonance imaging, citrate, choline, HR-MAS MRS, color-based segmentation

## Abstract

**Introduction:**

Magnetic resonance imaging (MRI) can portray spatial variations in tumor heterogeneity, architecture, and its microenvironment in a non-destructive way. The objective of this study was to assess the relationship between MRI parameters measured on patients *in vivo*, individual metabolites measured in prostatectomy tissue *ex vivo*, and quantitative histopathology.

**Materials and methods:**

Fresh frozen tissue samples (*n* = 53 from 15 patients) were extracted from transversal prostate slices and linked to *in vivo* MR images, allowing spatially matching of *ex vivo* measured metabolites with *in vivo* MR parameters. Color-based segmentation of cryosections of each tissue sample was used to identify luminal space, stroma, and nuclei.

**Results:**

Cancer samples have significantly lower area percentage of lumen and higher area percentage of nuclei than non-cancer samples (*p* ≤ 0.001). Apparent diffusion coefficient is significantly correlated with percentage area of lumen (ρ = 0.6, *p* < 0.001) and percentage area of nuclei (ρ = −0.35, *p* = 0.01). There is a positive correlation (ρ = 0.31, *p* = 0.053) between citrate and percentage area of lumen. Choline is negatively correlated with lumen (ρ = −0.38, *p* = 0.02) and positively correlated with percentage area of nuclei (ρ = 0.38, *p* = 0.02).

**Conclusion:**

Microstructures that are observed by histopathology are linked to MR characteristics and metabolite levels observed in prostate cancer.

## Introduction

Magnetic resonance imaging (MRI) plays an important role in the diagnostic work-up of prostate cancer patients (1). MRI can portray spatial variations in tumor heterogeneity, architecture, and its microenvironment in a non-destructive way. Metabolic and morphologic changes in prostate cancer tissue lead to changes in MRI and MR spectroscopy (MRS) parameters. In cancer areas of the prostate’s peripheral zone, T_2_ intensity and apparent diffusion coefficient (ADC) are reduced, while the choline and creatine-to-citrate ratio is increased compared to non-cancer areas (2, 3). These cancer-related changes in MR imaging parameters may be caused by a combination of increased cellularity, reduced luminal space, and altered metabolism.

Attempts have been made to elucidate the relationship between MR visible metabolites and MR imaging parameters. A negative correlation between ADC and the metabolite ratio choline plus creatine-to-citrate has been demonstrated (4). Due to low spectral resolution *in vivo*, correlation between MRI parameters and individual metabolites (such as choline-containing compounds and citrate) has not yet been investigated. *Ex vivo* high-resolution spectroscopy data from magic angle spinning (HR-MAS) MRS from prostate cancer can be linked to *in vivo* MR parameters as previously described (5), and individual metabolites can therefore be correlated with MRI parameters.

In the prostate, healthy peripheral zone has heterogeneous tissue architecture and consists primarily of glandular lumen lined with secretory epithelium embedded within a stromal matrix (6). Healthy prostate tissue is characterized by high levels of citrate since the glandular secretory epithelial cells have the ability to produce, accumulate, and secrete citrate (7). The water content is low in stroma, but high in luminal space resulting in a relatively long T_2_ and unrestricted water diffusion. As a result, healthy peripheral zone shows high signal intensity in T_2_-weighted images and on ADC maps, while T_2_ intensity and ADC are reduced in cancer (8–14). However, it has been demonstrated by Bourne et al. that the secretory epithelia within healthy peripheral zone represent a compartment of highly restricted water diffusion (6) and that healthy peripheral zone in general displays two T_2_ components, one liquid-like component with long T_2_ time originating from the luminal space and one component with a short T_2_ time originating from stromal and epithelial tissues (15). Further, significant correlations between imaging parameters and histological features, such as luminal space, cell density, percentage of nuclei, and cytoplasm, have been demonstrated (16–18).

The objective of this study was to assess the relationship between MRI parameters (T_2_ intensity and ADC) measured on patients *in vivo*, individual metabolites measured in prostatectomy tissue *ex vivo* and quantitative histopathological features (percentage nuclei, stroma, and luminal space). An overview of these relationships could give a better insight into the origin of the observed MRI and MRS signals and contribute to better understanding of the similarities and differences of these parameters.

## Materials and Methods

### Patients and Tissue Samples

The Regional Committee for Medical Research Ethics approved the study, and patients gave informed written consent to participate. Fresh frozen tissue samples [*n* = 53 from 15 patients, median 3 (range 2–6) per patient] were extracted from transversal prostate slices and linked to *in vivo* MR parameters as previously described (5, 19). In short, a full transversal, fresh tissue slice (2 mm thick) was resected from the middle of the prostate and snap-frozen. Tissue samples (3 mm in diameter) were thereafter drilled out of the frozen tissue slice, and locations of removed samples were documented on a photo. Preoperative MR images best corresponding to the level of the resected tissue slice were identified, and circular regions of interest were outlined according to the location of the removed tissue samples. Patient and tissue sample characteristics are listed in Table 1.

**Table 1:** **Characteristics of patients (*N* = 15) and tissue samples (*n* = 53)**.

**Patient characteristics**	**Value, median (range)**
Age (years)	63.7 (48.0–69.5)
sPSA (ng/ml)	12.0 (5.9–21.4)
**Tissue sample characteristics**	***n***
Peripheral zone	33
Transition zone	20
Gleason score	
Non-cancer	14
6 (3 + 3)	11
7 (3 + 4, 4 + 3)	12 (8, 4)
8 (3 + 5, 4 + 4)	8 (2, 6)
9 (4 + 5, 5 + 4)	8 (5, 3)
Tumor load (%)^a^, median (range)	60 (10–90)

*sPSA, serum prostate-specific antigen at time of surgery; *n* = number of patients tissue samples*.

*^a^Percentage tumor in tumor-containing tissue samples*.

### MR Imaging

Magnetic resonance imaging was performed as previously described (2). In short, patients with biopsy proven prostate cancer underwent a preoperative multiparametric MR examination including T_2_-weighted imaging (T_2_WI), diffusion-weighted imaging (DWI), MR spectroscopic imaging [MRSI; results previously reported in Ref. (2, 5) and not shown here], and dynamic contrast-enhanced magnetic resonance imaging [DCE-MRI; results previously reported in Ref. (2) and not shown here] on a 3-T system (Magnetom Trio, Siemens Medical Solutions, Erlangen, Germany). Phased array body coil and spine coil elements were used for signal detection. T_2_-weighted turbo spin echo images were obtained in three orthogonal planes. The transversal T_2_-weighed images (TR/TE 4210 ms/104 ms, FOV 160 mm × 160 mm, matrix 320 × 256, slice thickness 3 mm, and acquisition time 5 min 47 s) were angulated perpendicular to the urethra to replicate the angle of slicing for histopathological analysis. Diffusion-weighted images [TR/TE 3500 ms/77 ms, FOV 340 mm × 168 mm, matrix 170 × 170, slice thickness 4 mm, four *b*-values (50, 300, 600, and 800 s/mm^2^), and acquisition time 2 min 59 s] and dynamic contrast-enhanced images (TR/TE 4 ms/1.34 ms, FOV 280 mm × 227.6 mm, matrix 256 × 230.4, slice thickness 2 mm, temporal resolution 12.9 s, and total acquisition time 5 min 32 s) were equally angulated. T_2_-weighted images and ADC maps were used to calculate T_2_ intensities and ADC in regions of interest corresponding to tissue resection areas.

### HR-MAS MRS Experiment

^1^H HR-MAS MR spectra of the tissue samples were obtained using a 14.1-T spectrometer (Bruker Avance DRX 600, Bruker BioSpin GmbH, Karlsruhe, Germany) and post-processed as previously described (20). Quantification of metabolites was performed by LC Model (21), as described by Giskeødegård et al. (20).

### Histopathology and Color-Based Segmentation

**Figure 1:**
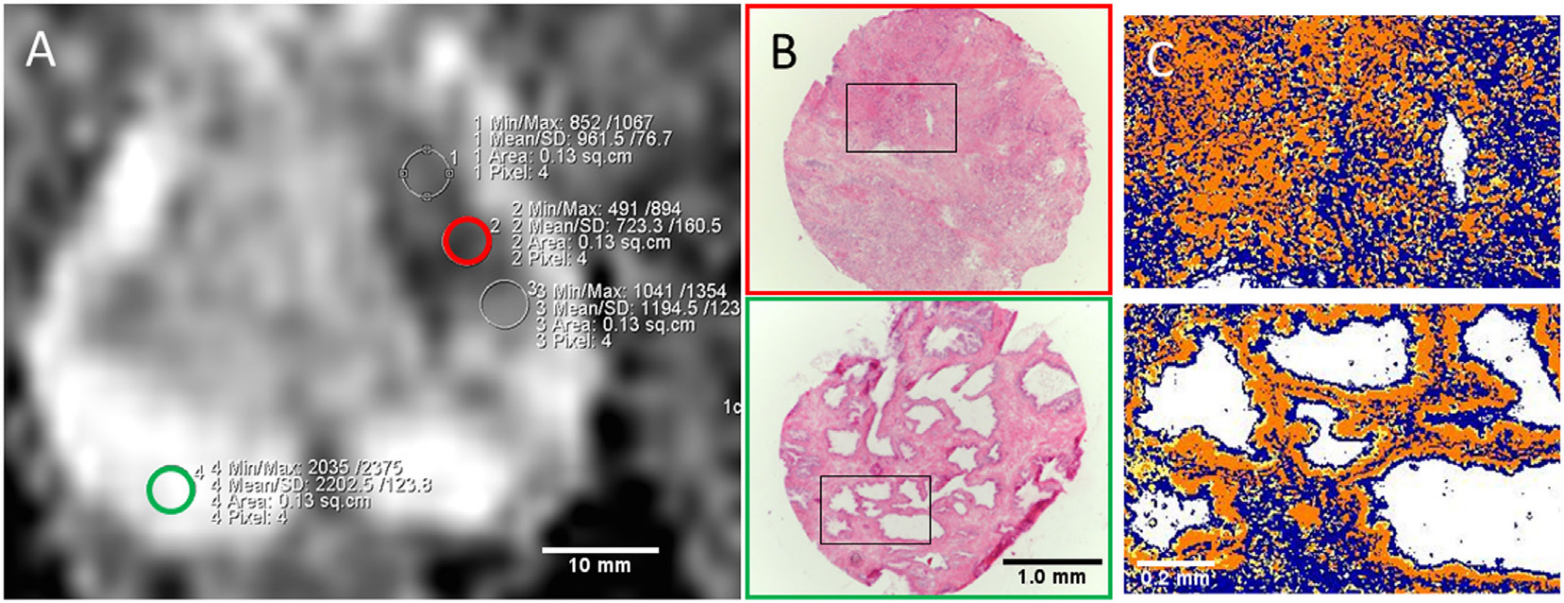
**(A)** ADC map with ROIs from cancer (red outline) and non-cancer peripheral zone (green outline) areas corresponding to extracted tissue samples. **(B)** H&E stained slides (4× objective) of cryosections with Gleason 5 + 4 (red) and non-cancer (green) tissue. Red and green outline refer to tissue location in **(A)**. **(C)** Close-up view of corresponding color-based segmentation with setting 2. Negative pixels (blue color) correspond to stroma; positive pixels (yellow and orange color) correspond to cytoplasm and nuclei; lumen is calculated as total number of pixels minus positive and negative (white color).

A cryosection was taken from each tissue sample and stained with hematoxylin and eosin (H&E). These H&E-stained slides were digitized with 4× magnification, and color-based segmentation (Positive Pixel Count algorithm in ImageScope v.11, Aperio Technologies) was used to identify luminal space, stroma, and nuclei, as described by Langer et al. (16). In short, two hue and windows settings were used (setting 1: 0.1 for hue, 0.5 for window; setting 2: 0.7 for hue, 0.35 for window) and optimized for each histologic slide by adjusting the window on a test region such that negative pixels in setting 1 represented nuclei, negative pixels in setting 2 represented stroma, and positive pixels in setting 2 represented cytoplasm and nuclei. Lumen was calculated as total area minus positive and negative pixels in setting 2 (Figure 1). For all the components (lumen, stroma, and nuclei), percentage of total area was used in the calculations. One tissue sample (GS 4 + 4) was excluded from color-based segmentation due to poor quality of the H&E slide. The metabolite concentrations from this tissue sample were included in the analyses when metabolites were correlated with MR parameters.

### Statistics

The Kolmogorov–Smirnov test was used to test data normality. Linear mixed model was used for pairwise comparison of histological components and different Gleason scores. Linear mixed model was also used to evaluate the association between MR parameters, metabolites, and histological features. Parameters that were not normally distributed were log-transformed before being entered in the linear mixed model. To account for multiple samples per patient, patient identification was entered as a random effect on the intercept in the model. Spearman’s rank correlation (ρ) was calculated between *in vivo* MR parameters, *ex vivo* metabolite concentrations, and histological features since they were not all normally distributed. This measure does not take into account multiple samples per patient. Multiple comparisons were corrected for with the Benjamini and Hochberg false discovery rate. Adjusted *p* values <0.05 were considered significant. All statistical analyses were performed using SPSS (IBM SPSS Statistics 22.0), except Benjamini and Hochberg corrections, which were performed in Matlab (MATLAB R2009a, The MathWorks Inc., Natick, MA, USA).

## Results

**Figure 2:**
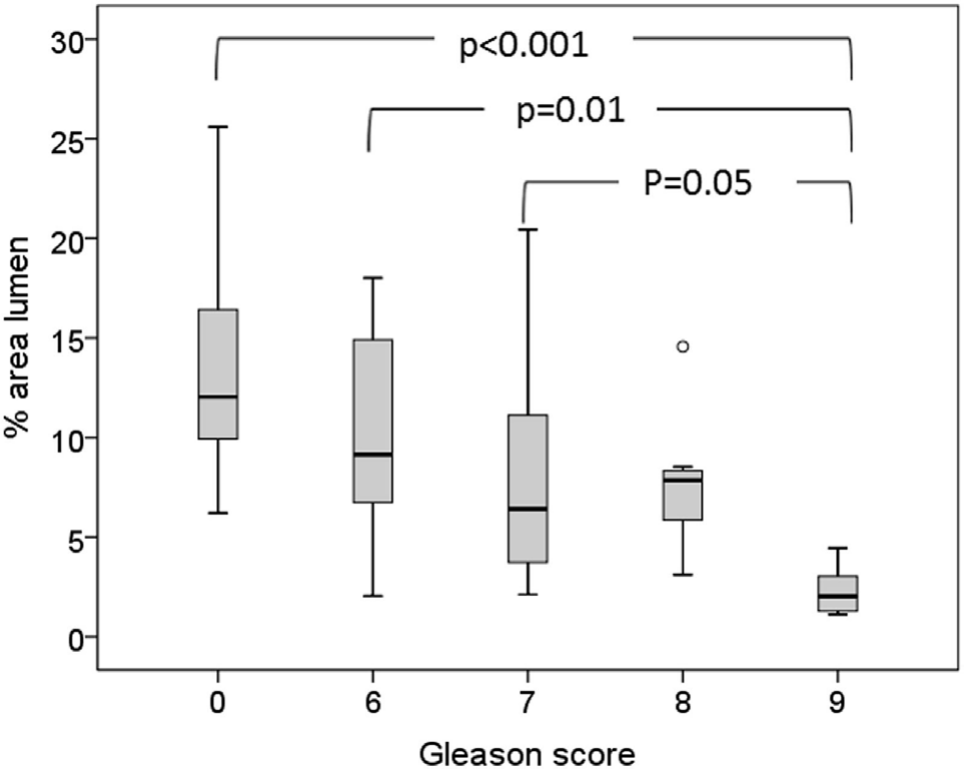
**Box-plot showing association between Gleason score and percentage area of lumen**.

An overview of histological components in cancer and non-cancer samples is given in Table 2. Cancer samples have significantly lower area percentage of lumen and higher area percentage of nuclei than non-cancer samples (*p* ≤ 0.001). Percentage area of stroma is not significantly different between cancer and non-cancer samples (*p* = 0.3). There are no significant differences between histological parameters in tissue samples with different Gleason scores except for percentage area of lumen in Gleason score 9 samples, which are significantly lower than for Gleason scores 6 and 7 (Figure 2).

**Table 2:** **Area percentage of histological components**.

	Lumen (%)	Stroma (%)	Nuclei (%)	No. of samples
**Tissue type**				
Non-cancer	13.8 ± 1.6	54.8 ± 1.9	23.3 ± 1.9	14
Cancer	**7.5 ± 0.9**	50.7 ± 1.2	**30.3 ± 1.0**	38
GS 6	10.4 ± 1.5	50.2 ± 1.7	28.5 ± 1.6	11
GS 7	**8.2 ± 1.7**	47.9 ± 2.1	**31.2 ± 2.0**	12
GS 8	**7.7 ± 1.4**	52.0 ± 3.1	**31.5 ± 2.4**	7
GS 9	**2.3 ± 0.4**	54.4 ± 3.1	**30.5 ± 2.2**	8

*Numbers are mean ± SE. Bold font indicates a significant difference from non-cancer samples (*p* < 0.05 after correction for multiple comparisons)*.

The *in vivo*-measured MR parameters, T_2_ intensity and ADC, are reduced in areas of cancer compared to non-cancer (Table 3) (*p* < 0.001). There is a trend toward reduced ADC and T_2_ intensity with higher Gleason score and a significantly lower ADC in Gleason score 9 samples compared to Gleason score 6 samples (*p* = 0.02). The *ex vivo*-measured metabolite concentrations of citrate and choline is reduced and increased, respectively, in cancer samples compared to non-cancer samples (Table 3). There is a trend toward increased choline and decreased citrate with increased Gleason score and significantly lower citrate in Gleason score 9 samples compared to Gleason score 6 samples (*p* = 0.01). There are no significant differences in choline between samples with different Gleason scores.

**Table 3:** **MR parameters**.

	ADC (×10^**−**6^ mm^2^/s)	T_2_ intensity	Choline^a^ (mmol/kg^b^)	Citrate (mmol/kg^b^)	No. of samples
**Tissue type**					
Non-cancer	1572 ± 95	353 ± 31	1.19 ± 0.1	9.8 ± 1.4	14
Cancer	**1146 ± 44**	**250 ± 11**	**2.8 ± 0.3**	**6.6 ± 0.7**	39
GS 6	**1265 ± 97**	281 ± 18	**2.0 ± 0.5**	9.7 ± 1.4	11
GS 7	**1164 ± 70**	**254 ± 22**	**3.0 ± 0.5**	**5.4 ± 1.1**	12
GS 8	**1180 ± 88**	**232 ± 21**	**2.8 ± 0.5**	**7.1 ± 1.6**	8
GS 9	**921 ± 60**	**220 ± 21**	**3.7 ± 0.8**	**3.6 ± 0.9**	8

*Numbers are mean ± SE. Bold font indicates a significant difference from non-cancer samples (*p* < 0.05 after correction for multiple comparisons)*.

*^a^Sum of all choline-containing compounds*.

*^b^Choline and citrate concentrations are reported as millimoles per kilogram wet weight. T_2_ intensity, total choline, and citrate levels were not normally distributed and thus were log-transformed before being entered into the linear mixed model*.

**Figure 3:**
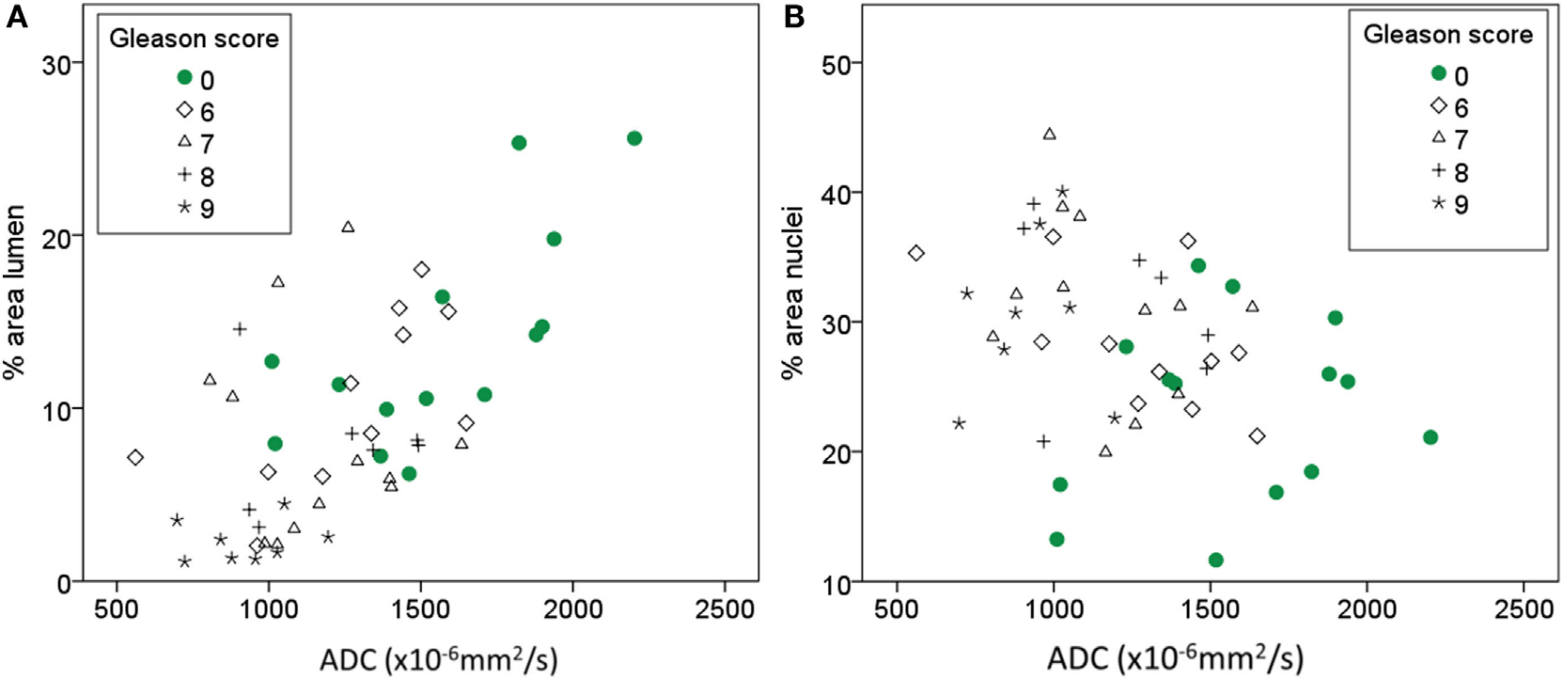
**Scatterplot showing the relationship between ADC and percentage area of lumen (A) and percentage area of nuclei (B), respectively**.

There is an intermediate to strong positive correlation between ADC and percentage area of lumen (ρ = 0.6, *p* < 0.001). There is a weaker, but significant, negative correlation between ADC and percentage area of nuclei (ρ = −0.35, *p* = 0.01) (Table 4; Figure 3). ADC, percentage area of lumen, and percentage area of nuclei are all correlated with Gleason score in the tissue sample (ρ = −0.58, *p* < 0.001; ρ = −0.62, *p* < 0.001; and ρ = 0.4, *p* = 0.01, respectively) (Table 4). When only non-cancer samples (*n* = 14) are considered, there is still a strong correlation between ADC and lumen (ρ = 0.7, *p* = 0.005), while the correlation between ADC and nuclei is no longer significant (ρ = −0.007, *p* = 0.982). In linear mixed model analysis, percentage area of lumen, nuclei, and Gleason score are significant covariates of ADC (*p* < 0.001, *p* = 0.014, and *p* < 0.001, respectively). Percentage area of stroma is not significantly correlated with ADC or Gleason score (Table 4).

**Table 4:** **Correlation coefficients between MR parameters and histological features**.

	Lumen (%)	Nuclei (%)	Stroma (%)	Gleason score
ADC (×10^−6^ mm^2^/s)	**0.60** (<0.001)	−**0.35** (0.03)	−0.01 (0.99)	−**0.58** (<0.001)
Citrate (mmol/kg^a^)	0.31 (0.05)	0.09 (0.71)	−0.01 (0.99)	−**0.46** (0.002)
Choline (mmol/kg^a^)	−**0.38** (0.01)	**0.38** (0.01)	−0.19 (0.31)	**0.55** (<0.001)
Spermine (mmol/kg^a^)	0.18 (0.37)	0.12 (0.67)	0.004 (0.99)	−0.31 (0.05)
Creatine (mmol/kg^a^)	−0.09 (0.71)	0.07 (0.57)	0.08 (0.71)	−0.07 (0.71)
(cho + spm + cre)/cit	−**0.49** (0.001)	0.07 (0.71)	0.003 (0.99)	**0.60** (<0.001)
(cit + spm + cre)/cho	**0.45** (0.003)	−0.22 (0.24)	0.13 (0.55)	−**0.69** (<0.001)
Gleason score	−**0.62** (<0.001)	**0.40** (0.01)	−0.14 (0.51)	

*Denoted in brackets are the *p*-values corrected for multiple testing with Benjamini and Hochberg false discovery rate approach*.

*cho, choline; spm, spermine; cre, creatine; cit, citrate*.

Bold font indicates a significant correlation (<0.05 after correction from multiple comparisons)

*^a^Metabolite concentrations are reported as millimoles per kilogram wet weight*.

**Figure 4:**
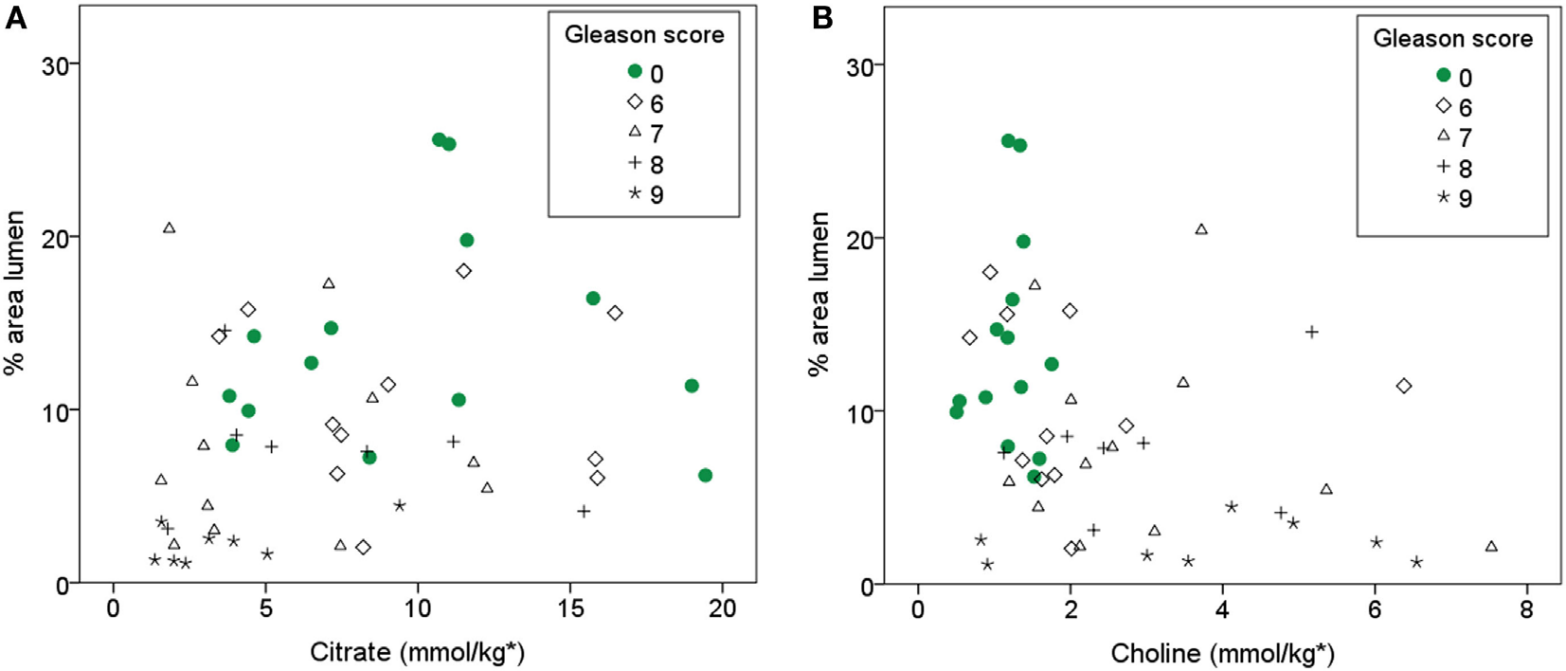
**Scatterplot showing the relationship between percentage area of lumen and citrate (A) and choline (B), respectively**. *Citrate and choline concentrations are reported as millimoles per kilogram wet weight.

There is a positive correlation (ρ = 0.31) between Citrate and percentage area of lumen, however after correcting for multiple testing, it is only borderline significant at the 0.05 level (*p* = 0.053). With linear mixed model, lumen is a significant covariate of citrate (*p* = 0.005). Choline is negatively correlated with lumen (ρ = −0.38, *p* = 0.02) and positively correlated with percentage area of nuclei (ρ = 0.38, *p* = 0.02). Both citrate and choline are significantly correlated with Gleason score (ρ = −0.46, *p* = 0.002; ρ = 0.55, *p* < 0.001, respectively). When only non-cancer samples are considered (*n* = 14) citrate is not significantly correlated with percentage area of lumen (ρ = 0.15, *p* = 0.65) and choline is not significantly correlated with lumen or nuclei (ρ = 0.01, *p* = 0.95; ρ = 0.24, *p* = 0.41, respectively). The relationship between percentage area of lumen, citrate, choline, and Gleason score is visualized in Figure 4. Spermine and creatine are not significantly correlated with any of the glandular components, but the ratios (choline + spermine + creatine)/citrate and (citrate + spermine + creatine)/choline are significantly correlated with percentage area of lumen (ρ = 0.49, *p* = 0.002; ρ = −0.45, *p* = 0.004, respectively) (Table 4).

## Discussion

In this study, we have explored the relationship between MR parameters measured *in vivo* (ADC and T_2_ intensity), metabolite concentrations (citrate, choline, creatine, and spermine) measured *ex vivo* with HR-MAS MRS, and histological gland components (percentage area of lumen, nuclei, and stroma) extracted from histopathology with color-based segmentation.

As expected, we find a clear difference in histological features between cancer and non-cancer samples. The decrease in percentage of luminal space and increase in percentage area of nuclei in cancer samples compared to non-cancer samples is in line with previous reported findings by several groups (16, 22, 23). The percentage area of lumen in healthy prostate tissue has previously been reported to be in the range of 20–30% (16, 22), which is higher than we observed. Previous studies have performed color-based segmentation on H&E-stained whole mount sections while we used H&E-stained cryosections. H&E staining of cryosections can be of lower quality than staining of paraffin-embedded tissue, and this might explain some of the discrepancy. Further, our samples were 3 mm in diameter, and luminal space on the edges was omitted in the segmentation, which could also result in lower values. However, we observed a trend toward decreased percentage area of lumen with increased Gleason score, consistent with previous findings (16, 23). The literature is inconsistent regarding stroma content in cancer versus non-cancer tissue (16, 22, 23); however, there was no evidence of differences in percentage area of stroma in our data.

We find a significantly increased percentage area of nuclei in cancer samples compared to non-cancer samples. In cancer samples, we see a weak trend toward increased area of nuclei with increased Gleason score; however, there is no significant difference between different Gleason scores. A trend toward increasing cellularity metrics with increasing Gleason pattern is described in numerous articles, but most of them lack significant differences between Gleason groups (16, 17, 22–24). This has often been attributed to the limited range of Gleason scores in the study cohorts. However, in our study, we have tissue samples ranging from non-cancer to Gleason score 9 with approximately equal number of samples in each group and still find no significant differences. This might be a result of the nature of the Gleason scoring system, where each pattern is based on tissue architecture rather than cellularity *per se*. This finding might also explain the lack of significant differences in choline between different Gleason scores, although there is a positive correlation between choline and percentage area of nuclei (ρ = 0.38, *p* = 0.021) in our data.

The basis for the widespread use of MR imaging in prostate cancer diagnostics is the reduced T_2_ intensity and ADC in cancer compared to healthy prostate (2, 3, 9), which this study also confirms. The observed trend toward decreased ADC and T_2_ intensity with increased Gleason score is also in line with previous findings (25, 26). It is likely that observed changes in tissue composition in cancer compared to non-cancer areas are responsible for the observed changes in MRI parameters. A more detailed investigation of the correlations between MR parameters and glandular components could help us to understand what underlying structural changes have the strongest effect on the observed changes in the MR images. Bourne et al. highlight that there are three diffusion compartments in prostate tissue: ductal lumen with close to free diffusion, stromal tissue with intermediate diffusion, and epithelium with highly restricted diffusion (6). In our data, we see a positive correlation between ADC and percentage area of lumen. Since there is also a trend toward decreasing ADC with increasing Gleason score, we also tested the correlation between ADC and lumen in the non-cancer samples with a strong significant correlation also in that cohort. The percentage area of nuclei was negatively correlated with ADC; however, there was no correlation between ADC and nuclei when only non-cancer samples were investigated. Combined, these results indicate that in low resolution clinical DW images, the amount of luminal space is the main contributor to the measured ADC value, rather than increased cellularity, which is often used as the default explanation for reduced ADC in cancer imaging.

Altered tissue metabolism is an emerging hallmark of cancer (27). Although it is likely that metabolites that are stored in the luminal space, such as citrate and spermine, will be reduced when the lumen is invaded by cancer tissue, the observed change in metabolites between cancer and non-cancer tissue could also be due to cancer-related changes in metabolism. In our cohort, both citrate and luminal space were decreasing with increasing Gleason score. A weak correlation between citrate and luminal space was observed, but there was not a significant correlation when only normal samples were investigated. This could be due to the low number of non-cancer samples; however, it could also indicate that the correlation between citrate and luminal space is an indirect effect of their association with Gleason score. This could further indicate that the changes in metabolites, seen in cancer on MRSI, are not solely dependent on tissue composition and that citrate and choline measurements from MRSI give complementary information to ADC and T_2_WI.

*In vivo* MRSI has lower spectral resolution than *ex vivo* HR-MAS MRS, and metabolite ratios, rather than individual metabolites, are therefore often reported for MRSI studies. The ratio of choline, polyamines (mainly spermine), and creatine-to-citrate is often used, since the resonances of choline, polyamines, and creatine can be difficult to separate in a reliable way (2). We expected to see a better correlation between individual metabolites and glandular features than by using metabolite ratios; however, this was not the case. The metabolites citrate and spermine are both stored in the prostatic fluid in the lumen, and it would therefore make sense to calculate a metabolite ratio where citrate and spermine are added. Kobus et al. (22) focused on the metabolite ratio citrate, spermine, and creatine-to-choline and found a positive correlation with lumen with correlation coefficient of 0.5. We find a correlation in the same range between this ratio and percentage area of lumen (ρ = 45), even though the individual metabolites are weaker correlated with lumen.

This study has some limitations. The low number of tissue samples hampered subdivision into peripheral zone and transition zone samples. There are also some inherent difficulties in the matching between *in vivo* MR images and tissue samples resected for HR-MAS NMR experiments and histopathology; however, the tissue harvesting method used in this study contributes to minimize the matching uncertainty (19). Previous studies have shown that there is a good correlation between *in vivo* and *ex vivo* measured metabolite ratios by using the harvesting and matching technique used in this study (5).

The acquired T_2_-weighted MR images did not allow for quantitative T_2_ measurements, and we therefore used T_2_ intensity, rather than T_2_ relaxation, in our analysis. Since T_2_ intensity can be affected by other factors than pure T_2_ relaxation, we chose to only do brief descriptive statistics of the T_2_ intensity. Further for ADC calculations, we used a monoexponential diffusion model, since this is the model which is available for ADC calculation in our clinical scanners. The monoexponential diffusion model assumes Gaussian diffusion conditions. This could affect the correlation between ADC and percentage area lumen, since the lumen contains freely diffusing liquid. Bourne et al. suggest that the distinct stromal and glandular diffusion compartments are the origin of the biexponential diffusion decay seen *in vivo* (6). Therefore, application of a biexponential diffusion model might have resulted in a higher correlation of ADC with area of nuclei or stroma.

All samples in this study were taken from patients with prostate cancer. The non-cancer samples were taken from a transversal prostate slice that contained prostate cancer elsewhere, and it is not yet fully understood how the metabolism in normal-appearing tissue is affected by the adjacent cancer (field effect). The citrate production in the histopathologically normal-appearing areas could already have been altered and therefore preclude the expected correlation between citrate and percentage of lumen in non-cancer samples.

## Conclusion

This study adds to the literature of associations between alterations in tissue composition, metabolism, and observed MR imaging parameters. The microstructures that are observed by histopathology are linked to MR characteristics in prostate cancer, and ADC appears to mainly reflect luminal space rather than dense tumor structures.

## Author Contributions

KS: study design, data collection, data analysis and interpretation, drafting the manuscript, and critical revision of the manuscript. RV: data analysis and interpretation, drafting the manuscript, and critical revision of the manuscript. HB: data collection and critical revision of the manuscript. AW: data analysis and interpretation and critical revision of the manuscript. AH and AA: study design and critical revision of the manuscript. M-BT: study design, data collection, data analysis and interpretation, and critical revision of the manuscript. TB: study design, data analysis and interpretation, and critical revision of the manuscript.

## Conflict of Interest Statement

The authors declare that the research was conducted in the absence of any commercial or financial relationships that could be construed as a potential conflict of interest.

## References

[1] Heidenreich A Bastian PJ Bellmunt J Bolla M Joniau S van der Kwast T EAU guidelines on prostate cancer. Part 1: screening, diagnosis, and local treatment with curative intent-update 2013. Eur Urol (2014) 65(1):124–37. 10.1016/j.eururo.2013.09.046 24207135

[2] Selnæs KM Heerschap A Jensen LR Tessem MB Schweder GJ Goa PE Peripheral zone prostate cancer localization by multiparametric magnetic resonance at 3 T: unbiased cancer identification by matching to histopathology. Invest Radiol (2012) 47(11):624–33. 10.1097/RLI.0b013e318263f0fd 23011187

[3] Hoeks CM Barentsz JO Hambrock T Yakar D Somford DM Heijmink SW Prostate cancer: multiparametric MR imaging for detection, localization, and staging. Radiology (2011) 261(1):46–66. 10.1148/radiol.11091822 21931141

[4] Kobus T Vos PC Hambrock T De Rooij M Hulsbergen-van de Kaa CA Barentsz JO Prostate cancer aggressiveness: in vivo assessment of MR spectroscopy and diffusion-weighted imaging at 3 T. Radiology (2012) 265(2):457–67. 10.1148/radiol.12111744 22843767

[5] Selnæs KM Gribbestad IS Bertilsson H Wright A Angelsen A Heerschap A Spatially matched in vivo and ex vivo MR metabolic profiles of prostate cancer – investigation of a correlation with Gleason score. NMR Biomed (2013) 26(5):600–6. 10.1002/nbm.2901 23280546

[6] Bourne R Kurniawan N Cowin G Sved P Watson G. 16 T diffusion microimaging of fixed prostate tissue: preliminary findings. Magn Reson Med (2011) 66(1):244–7. 10.1002/mrm.22778 21695726

[7] Costello LC Franklin RB Citrate metabolism of normal and malignant prostate epithelial cells. Urology (1997) 50(1):3–12. 10.1016/S0090-4295(97)00124-6 9218011

[8] Hegde JV Mulkern RV Panych LP Fennessy FM Fedorov A Maier SE Multiparametric MRI of prostate cancer: an update on state-of-the-art techniques and their performance in detecting and localizing prostate cancer. J Magn Reson Imaging (2013) 37(5):1035–54. 10.1002/jmri.23860 23606141 PMC3741996

[9] deSouza NM Reinsberg SA Scurr ED Brewster JM Payne GS. Magnetic resonance imaging in prostate cancer: the value of apparent diffusion coefficients for identifying malignant nodules. Br J Radiol (2007) 80(950):90–5. 10.1259/bjr/24232319 17303616

[10] Hosseinzadeh K Schwarz SD. Endorectal diffusion-weighted imaging in prostate cancer to differentiate malignant and benign peripheral zone tissue. J Magn Reson Imaging (2004) 20(4):654–61. 10.1002/jmri.20159 15390142

[11] Issa B. In vivo measurement of the apparent diffusion coefficient in normal and malignant prostatic tissues using echo-planar imaging. J Magn Reson Imaging (2002) 16(2):196–200. 10.1002/jmri.10139 12203768

[12] Kozlowski P Chang SD Jones EC Berean KW Chen H Goldenberg SL Combined diffusion-weighted and dynamic contrast-enhanced MRI for prostate cancer diagnosis – correlation with biopsy and histopathology. J Magn Reson Imaging (2006) 24(1):108–13. 10.1002/jmri.20626 16767709

[13] Mazaheri Y Shukla-Dave A Hricak H Fine SW Zhang J Inurrigarro G Prostate cancer: identification with combined diffusion-weighted MR imaging and 3D ^1^H MR spectroscopic imaging – correlation with pathologic findings. Radiology (2008) 246(2):480–8. 10.1148/radiol.2462070368 18227542

[14] Sato C Naganawa S Nakamura T Kumada H Miura S Takizawa O Differentiation of noncancerous tissue and cancer lesions by apparent diffusion coefficient values in transition and peripheral zones of the prostate. J Magn Reson Imaging (2005) 21(3):258–62. 10.1002/jmri.20251 15723379

[15] Storås TH Gjesdal KI Gadmar ØB Geitung JT Kløw NE. Prostate magnetic resonance imaging: multiexponential T_2_ decay in prostate tissue. J Magn Reson Imaging (2008) 28(5):1166–72. 10.1002/jmri.21534 18972358

[16] Langer DL van der Kwast TH Evans AJ Plotkin A Trachtenberg J Wilson BC Prostate tissue composition and MR measurements: investigating the relationships between ADC, T_2_, K_trans_, v_e_, and corresponding histologic features. Radiology (2010) 255(2):485–94. 10.1148/radiol.10091343 20413761

[17] Gibbs P Liney GP Pickles MD Zelhof B Rodrigues G Turnbull LW. Correlation of ADC and T_2_ measurements with cell density in prostate cancer at 3.0 Tesla. Invest Radiol (2009) 44(9):572–6. 10.1097/RLI.0b013e3181b4c10e 19692841

[18] Wang XZ Wang B Gao ZQ Liu JG Liu ZQ Niu QL Diffusion-weighted imaging of prostate cancer: correlation between apparent diffusion coefficient values and tumor proliferation. J Magn Reson Imaging (2009) 29(6):1360–6. 10.1002/jmri.21797 19472393

[19] Bertilsson H Angelsen A Viset T Skogseth H Tessem MB Halgunset J. A new method to provide a fresh frozen prostate slice suitable for gene expression study and MR spectroscopy. Prostate (2011) 71(5):461–9. 10.1002/pros.21260 20860008

[20] Giskeødegård GF Bertilsson H Selnæs KM Wright AJ Bathen TF Viset T Spermine and citrate as metabolic biomarkers for assessing prostate cancer aggressiveness. PLoS One (2013) 8(4):e62375. 10.1371/journal.pone.0062375 23626811 PMC3633894

[21] Provencher SW. Estimation of metabolite concentrations from localized in vivo proton NMR spectra. Magn Reson Med (1993) 30(6):672–9. 10.1002/mrm.1910300604 8139448

[22] Kobus T van der Laak JA Maas MC Hambrock T Bruggink CC Hulsbergen-van de Kaa CA Contribution of histopathologic tissue composition to quantitative MR spectroscopy and diffusion-weighted imaging of the prostate. Radiology (2016) 278(3):801–11. 10.1148/radiol.2015142889 26418614

[23] Chatterjee A Watson G Myint E Sved P McEntee M Bourne R. Changes in epithelium, stroma, and lumen space correlate more strongly with gleason pattern and are stronger predictors of prostate ADC changes than cellularity metrics. Radiology (2015) 277(3):751–62. 10.1148/radiol.2015142414 26110669

[24] Zelhof B Pickles M Liney G Gibbs P Rodrigues G Kraus S Correlation of diffusion-weighted magnetic resonance data with cellularity in prostate cancer. BJU Int (2009) 103(7):883–8. 10.1111/j.1464-410X.2008.08130.x 19007373

[25] Wang L Mazaheri Y Zhang J Ishill NM Kuroiwa K Hricak H. Assessment of biologic aggressiveness of prostate cancer: correlation of MR signal intensity with Gleason grade after radical prostatectomy. Radiology (2008) 246(1):168–76. 10.1148/radiol.2461070057 18024440

[26] Hambrock T Somford DM Huisman HJ van Oort IM Witjes JA Hulsbergen-van de Kaa CA Relationship between apparent diffusion coefficients at 3.0-T MR imaging and Gleason grade in peripheral zone prostate cancer. Radiology (2011) 259(2):453–61. 10.1148/radiol.11091409 21502392

[27] Hanahan D Weinberg RA Hallmarks of cancer: the next generation. Cell (2011) 144(5):646–74. 10.1016/j.cell.2011.02.013 21376230

